# Fc Engineering Approaches to Enhance the Agonism and Effector Functions of an Anti-OX40 Antibody*

**DOI:** 10.1074/jbc.M116.757773

**Published:** 2016-11-17

**Authors:** Di Zhang, Monica V. Goldberg, Mark L. Chiu

**Affiliations:** From Janssen Research and Development, L.L.C., Spring House, Pennsylvania 19477

**Keywords:** antibody engineering, anticancer drug, Fc receptor, immunotherapy, tumor necrosis factor (TNF), ADCC, OX40, agonism

## Abstract

Agonistic antibodies directed against immunostimulatory receptors belonging to the tumor necrosis factor receptor (TNFR) superfamily are emerging as promising cancer immunotherapies. Several Fc engineering approaches discovered recently can augment the anti-tumor activities of TNFR antibodies by enhancing their agonistic activities and/or effector functions. In this study, we compared these approaches for their effects on an anti-OX40 antibody. Both S267E/L328F and V12 mutations facilitated enhanced binding to FcγRIIB and thus increased FcγRIIB cross-linking mediated agonist activity. However, both mutations abrogated the binding to FcγRIIIA and thereby decreasing the antibody-dependent cellular cytotoxicity activities. In contrast, the E345R mutation, which can promote antibody multimerization upon receptor binding, facilitated anti-OX40 antibody to have increased agonism by promoting the clustering of OX40 receptors without the dependence on FcγRIIB cross-linking. Nonetheless, cross-linking to FcγRIIB can lead to a further boost of the agonism of the anti-OX40 antibody with IgG1 Fc but not with the silent IgG2σ Fc. The antibody-dependent cellular cytotoxicity and complement-dependent cytotoxicity activities of the anti-OX40 antibody with the E345R mutation were affected by the choice of IgG subtypes. However, there was little change in the antibody-dependent cellular phagocytosis activity. In summary, different Fc engineering approaches can guide the design of engineered antibodies to OX40 and other TNFR with improved anti-tumor activity.

## Introduction

Monoclonal antibodies that stimulate antitumor immunity are emerging as an important class of cancer therapeutics (1, 2). The antibodies (Abs) targeting the immune checkpoint receptors CTLA-4 and PD-1 have been approved as monotherapies for advanced melanoma, lung cancer, and evaluated for the treatment of other types of human cancer. Besides targeting the inhibitory pathways, agonist antibodies directed against the immunostimulatory receptors on T cells and antigen presenting cells can also stimulate antitumor immunity and are emerging as a promising area of clinical development for cancer immunotherapies (3).

Many immunostimulatory receptors belong to the tumor necrosis factor receptor superfamily (TNFRSF).^3^ TNFRSF include OX40, CD27, 4-1BB, HVEM, and GITR, which are expressed on effector T cells. The respective ligands and agonist antibodies can activate these receptors to stimulate the proliferation and activation of T cells (4–7). The activation of CD40 that is expressed on antigen presenting cells facilitates more efficacious presentation of tumor antigens to activated T cells (8, 9). Much evidence demonstrated the agonistic activities of therapeutic antibodies to TNFRSF are important for their anti-tumor activities (9–11). On the other hand, several TNFRSFs, such as OX40 and GITR, have elevated expression on regulatory T cells (T_reg_), which negatively modulate tumor immunity (12, 13). Several studies have revealed that the anti-OX40 and anti-GITR antibodies may facilitate the selective elimination of regulatory T cells in the tumor microenvironment by the effector functions of the antibody (12, 13). Such antibody-mediated killing of regulatory T cells may be more important than the antibody-mediated activation of effector T cells for the anti-tumor activities of therapeutic anti-OX40 and anti-GITR antibodies.

Accumulating evidence indicates that immunomodulatory antibodies engage different types of Fc receptors for their agonistic activities and effector functions. To activate downstream signaling pathways, receptor oligomerization is a prerequisite for TNFRSFs. Despite having bivalency, one antibody molecule may not be enough to cluster enough TNFRs. Instead, antibody cross-linking via attachment on beads or surface of assay plate can be necessary for receptor activation in *in vitro* assays (14). Recent studies in mice indicated that the engagement to the inhibitory FcγRIIB receptor is critical for the agonistic activity of antibodies to a number of TNFR targets, including CD40 (15, 16), death receptor 5 (DR5) (11, 17), and CD95 (18). The cross-linking of IgG Fc to FcγRIIB receptors can multimerize more than one antibody molecule, which in turn can facilitate the clustering of enough TNFRs for signaling pathway activation. On the other hand, the antibody effector functions, such as antibody-dependent cellular cytotoxicity (ADCC) and antibody-dependent cellular phagocytosis (ADCP), depend on the interactions with various activating Fcγ receptors. Studies in mice revealed that activating Fcγ receptors contribute to the antitumor activities of immunomodulatory anti-OX40 and anti-GITR antibodies by selectively eliminating intratumoral regulatory T cells (12, 13).

Unfortunately, human IgG antibodies have poor binding affinities to the majority of human Fc receptors except FcγRI (19). To optimize the antitumor activity of agonist antibodies for immunostimulatory TNFRSFs, one approach is to engineer the Fc region of the IgG antibody to improve its Fcγ receptor engagement, particularly through the engagement with FcγRIIB receptor, which mediates the agonism of TNFR antibodies. In this regard, Chu *et al.* (20) described S267E/L328F (serine at position 267 replaced with glutamic acid and leucine at position 328 replaced with phenylalanine) mutations in human IgG1 Fc domain with enhanced FcγRIIB binding affinity. Anti-CD19 antibody engineered with such mutations showed improved inhibition of B cell receptor-mediated activation of primary human B cells. However, further study revealed that such Fc variant also has enhanced binding to Arg^131^ allotype of the activating FcγRIIA receptor (21). Recently, Mimoto *et al.* (21) reported a set of six mutations in IgG1 Fc, collectively named as the V12 mutations, with selectively enhanced FcγRIIB engagement without associated increased binding to either His^131^ or Arg^131^ allotype of FcγRIIA receptor. The anti-CD137 antibody with the engineered V12 mutations showed much enhanced agonistic activity dependent on FcγRIIB engagement.

Although optimizing FcγRIIB engagement is a viable approach, the agonistic activity of such engineered antibodies depend heavily on the Fcγ receptor expression in the local microenvironment and the efficacy of such antibody may be limited to the anatomical site of action. In an effort to augment the agonism of immunostimulatory antibodies independent of Fcγ receptor engagement, White *et al.* (22) recently reported that human IgG2 hinge framework can impart superagonistic activity to immunostimulatory antibodies that target CD40, 4-1BB, and CD28 receptors. This activity is conferred by a unique configuration of disulfide bonds in the hinge region of the IgG2 subtype and is not dependent on FcγRIIB engagement. On the other hand, if the purpose of cross-linking to FcγRIIB is solely to increase the clustering of agonistic antibodies for receptor activation, then we hypothesized that Fc mutations that can promote antibody multimerization may enhance the agonism of antibodies to TNFRSFs without the need for FcγRIIB cross-linking. Diebolder *et al.* (23) reported that selective Fc mutations can facilitate IgG antibody hexamer formation upon binding target proteins on a cell surface. Although it was reported that such IgG hexamer can greatly activate ADCC, complement-dependent cytotoxicity (CDC), and induce apoptosis (24), we hypothesize that another application can be that oligomerized antibodies to TNFRSFs can activate the receptors by promoting receptor clustering.

Although many of the Fc mutations for Abs have been published in disparate reports, we present in this study a systematic evaluation of different Fc engineering approaches on the enhancement of the agonism of an anti-OX40 antibody. Besides, the effects of Fc mutations on the ADCC, ADCP, and CDC effector functions of the engineered antibodies were also evaluated. Such study can guide the design of engineered antibodies to OX40 and other TNFRSFs for improved anti-tumor activity.

## Results

### 

#### 

##### Establishment of a NF-κB Reporter Assay for the Assessment of the Agonism of an Anti-OX40 Antibody

**FIGURE 1.**
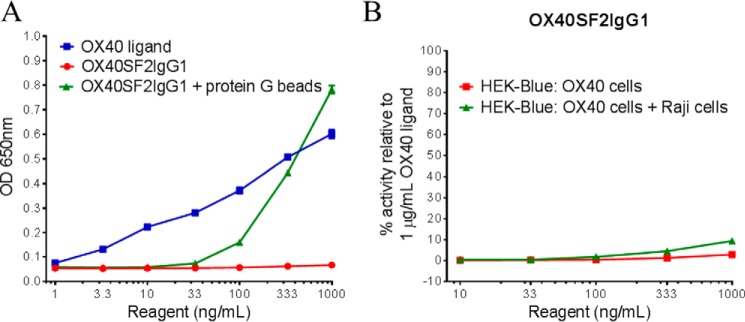
**HEK-Blue NF-κB reporter assay for the assessment of functional activities of OX40 ligand and anti-OX40 antibody.** *A,* increasing concentrations (1 to 1000 ng/ml) of OX40 ligand or OX40SF2IgG1 antibody in the absence or presence of protein G beads were incubated with HEK-Blue:OX40 cells and their agonistic activities were assessed by HEK-Blue NF-κB reporter assay. OD at 650 nm, which reflected the SEAP reporter gene expression levels, were plotted against the concentrations of test agents (Data are presented as mean ± S.E., *n* = 4). *B,* increasing concentrations (10 to 1000 ng/ml) of OX40SF2IgG1 antibody were incubated with HEK-Blue:OX40 cells with or without co-culturing with Raji cells and their agonistic activities were assessed by HEK-Blue NF-κB reporter assay. The agonistic activities of anti-OX40 antibodies, normalized as percent activity relative to that driven by 1 μg/ml of OX40 ligand, were plotted against the concentrations of test antibodies (Data were presented as mean ± S.E., *n* = 14).

OX40, a member 4 of TNFRSF (also known as CD134) activates the nuclear factor-κB (NF-κB) signaling pathway by binding to TNF receptor-associated factors (25, 26). To study the contributions of Fc engineering on the agonistic activity of OX40 antibodies, we established a HEK-Blue reporter cell line with stably expressing human OX40 that was used to set up a NF-κB reporter assay to assess OX40 functional activity. The reporter assay showed that OX40 ligand could activate the transfected OX40 receptor to elicit secreted embryonic alkaline phosphatase (SEAP) reporter gene expression in a dose-dependent manner (Fig. 1*A*). The OX40SF2IgG1, a humanized anti-OX40 antibody SF2 (27) with native IgG1 Fc, was then evaluated for its agonistic activity by this reporter assay. Although the monomeric antibody in solution showed little agonistic activity, OX40SF2IgG1 antibody immobilized on protein G beads could stimulate reporter gene expression in a dose-dependent manner and to a level better than OX40 ligand at 1000 ng/ml (Fig. 1*A*). This result demonstrated that antibody cross-linking was needed for the agonistic activity.

Recent studies revealed that FcγRIIB can provide the cross-linking activity and facilitate the agonistic activity of TNFR antibodies (28). The FcγRIIB cross-linking effect was assessed in the reporter assay by co-culturing HEK-Blue:OX40 cells with human B lymphoblastoid Raji cells that had FcγRIIB expression (29). However, co-culturing with Raji cells failed to significantly enhance the agonistic activity of SF2 antibody with native human IgG1 Fc domain (Fig. 1*B*).

##### Fcγ Receptor Binding Properties for Anti-OX40 Antibodies with S267E/L328F and V12 Mutations

**FIGURE 2.**
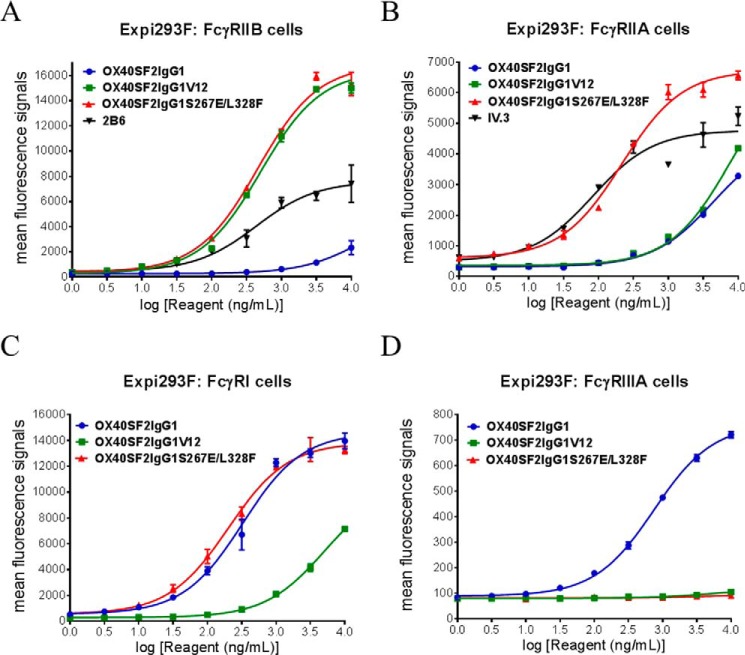
**Fcγ receptor binding properties for anti-OX40 antibodies with S267E/L328F and V12 mutations.** Increasing concentrations (1 to 10000 ng/ml) of OX40SF2IgG1, OX40SF2IgG1S267E/L328F, and OX40SF2IgG1V12 antibodies were assessed for their binding to Expi293F cells transfected with human FcγRIIB receptor (*A*), FcγRIIA receptor (*B*), FcγRI receptor (*C*), and FcγRIIIA receptor (*D*) by flow cytometry assays. Anti-FcγRII receptor antibodies 2B6 and IV.3 were also assessed for their binding to FcγRIIB receptor (*A*) and FcγRIIA receptor (*B*), respectively. Mean fluorescence signals were plotted against the concentrations of test antibodies (data were presented as mean ± S.E., *n* ≥ 2).

S267E/L328F and V12 mutations are Fc mutations that facilitate enhanced binding of human Ab to FcγRIIB receptor (20, 21). To evaluate the effects of such mutations on anti-OX40 antibody, we engineered OX40SF2IgG1 antibody to have the S267E/L328F double mutations (OX40SF2IgG1S267E/L328F) and the V12 mutations (OX40SF2IgG1V12). Their binding to Fcγ receptors expressed on transiently transfected Expi293F cells were assessed by flow cytometry. Although OX40SF2 antibody with native IgG1 had poor binding to FcγRIIB, the engineered Fc mutations potently increased EC_50_ values for OX40SF2IgG1S267E/L328F (459 ng/ml, 3.1 nm) and OX40SF2IgG1V12 (502 ng/ml, 3.4 nm) binding to FcγRIIB. These EC_50_ values were comparable with those for 2B6 (431 ng/ml, 2.9 nm), a monoclonal antibody with specific binding to FcγRIIB (30)(Fig. 2*A*). Similar flow cytometry assays were performed to assess the binding of engineered anti-OX40 antibodies to the Arg^131^ allotype of FcγRIIA expressed on transiently transfected Expi293F cells. Although OX40SF2IgG1V12 and OX40SF2IgG1 similarly had poor binding to FcγRIIA, the S267E/L328F mutations had a 20-fold more potent EC_50_ value (216 ng/ml, 1.5 nm) for binding to FcγRIIA^Arg131^ (Fig. 2*B*). This data corroborated previous findings that both S267E/L328F and V12 mutations could enhance FcγRIIB binding (21).

For FcγRI, OX40SF2IgG1 showed high affinity with an EC_50_ value of 326 ng/ml, 2.2 nm (Fig. 2*C*). The OX40SF2IgG1S267E/L328F antibody showed a similar binding property as OX40SF2IgG1. However, the V12 mutations significantly abrogated the binding to FcγRI. The binding of the engineered anti-OX40 antibodies to FcγRIIIA receptor were also evaluated by flow cytometry assay. Although OX40SF2IgG1 antibody had binding to FcγRIIIA (EC_50_ ∼ 744 ng/ml, 5.0 nm), the OX40SF2IgG1S267E/L328F and OX40SF2IgG1V12 Abs had no binding to FcγRIIIA (Fig. 2*D*).

##### S267E/L328F and V12 Mutations on Anti-OX40 Antibody Enhanced Agonism by Cross-linking to FcγRIIB

**FIGURE 3.**
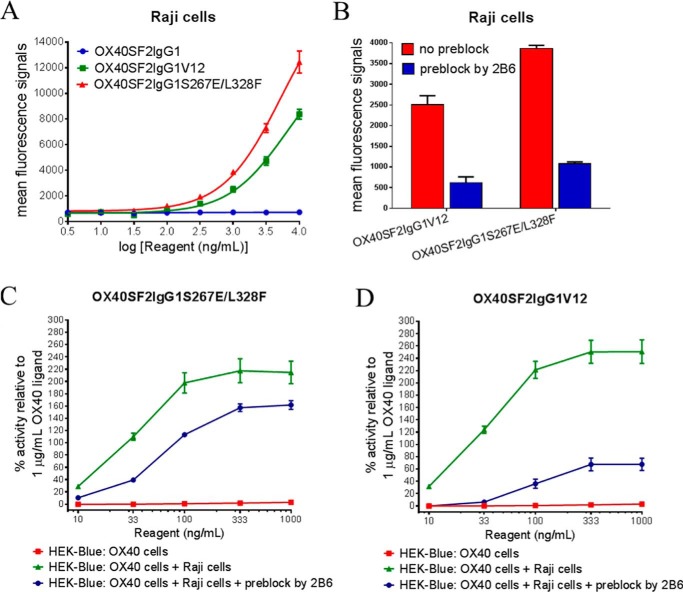
**Cross-linking to FcγRIIB receptors on Raji cells facilitated OX40SF2IgG1S267E/L328F and OX40SF2IgG1V12 antibodies with higher agonism.** *A,* increasing concentrations (3 to 10000 ng/ml) of OX40SF2IgG1, OX40SF2IgG1S267E/L328F, and OX40SF2IgG1V12 antibodies were assessed for their binding to Raji cells by flow cytometry assay. Mean fluorescence signals were plotted against the concentrations of test antibodies (data were presented as mean ± S.E., *n* = 3). *B,* Raji cells were pretreated with 5 μg/ml of 2B6 antibody and then the binding of 1 μg/ml of engineered anti-OX40 antibodies to Raji cells were assessed by flow cytometry. Mean fluorescence signals of binding without (*red*) and with (*blue*) preblock by 2B6 antibody were presented in the bar graphs as mean ± S.E., *n* = 3. *C* and *D,* increasing concentrations (10 to 1000 ng/ml) of OX40SF2IgG1S267E/L328F (*C*) and OX40SF2IgG1V12 (*D*) were incubated with HEK-Blue:OX40 cells with or without co-culturing with Raji cells. To test the contribution of FcγRIIB cross-linking, another set of assays were set up in which Raji cells were preincubated with 5 μg/ml of 2B6 antibody before co-culturing with HEK-Blue:OX40 cells. The agonistic activities of the antibodies were assessed by HEK-Blue NF-κB reporter assay. The agonistic activities of anti-OX40 antibodies, normalized as percent activity relative to that driven by 1 μg/ml of OX40 ligand, were plotted *versus* the concentrations of test antibodies (data were presented as mean ± S.E., *n* ≥ 6).

Because S267E/L328F and V12 mutations facilitate OX40SF2IgG1 antibody with increased binding affinity to FcγRIIB, we assessed whether these enhanced binding may lead to increased agonism of anti-OX40 antibody by the HEK-Blue NF-κB reporter assay. First, the binding of engineered anti-OX40 antibodies to Raji cells were assessed by a flow cytometry assay. OX40SF2IgG1S267E/L328F and OX40SF2IgG1V12, but not OX40SF2IgG1, showed a dose-dependent binding to Raji cells (Fig. 3*A*), albeit with less potency compared with Expi293F cells transfected with FcγRIIB receptor (Fig. 2*A*). To confirm the binding to Raji cells were mediated by FcγRIIB, we pre-treated Raji cells with 5 μg/ml of FcγRIIB-specific 2B6 antibody before assessing the binding of engineered anti-OX40 antibody to Raji cells. It was observed that 2B6 antibody significantly abrogated the binding of OX40SF2IgG1S267E/L328F and OX40SF2IgG1V12 to Raji cells (Fig. 3*B*).

In HEK-Blue NF-κB reporter assay, neither OX40SF2IgG1S267E/L328F nor OX40SF2IgG1V12 showed significant agonistic activity in the absence of Raji cells. However, with co-culturing Raji cells and HEK-Blue:OX40 cells, these engineered anti-OX40 antibodies showed dramatically increased agonism, with over 2-fold better efficacy compared with OX40 ligand at 1000 ng/ml (Fig. 3, *C* and *D*). When 2B6 antibody was added to pre-block FcγRIIB receptor on Raji cells, the Raji cell-dependent enhancement of agonism for OX40SF2IgG1S267E/L328F and OX40SF2IgG1V12 antibodies were significantly decreased (Fig. 3, *C* and *D*), suggesting that the agonistic activities of the engineered antibody were mediated by FcγRIIB cross-linking.

##### Fc Effector Functions for Anti-OX40 Antibodies with S267E/L328F and V12 Mutations

**FIGURE 4.**
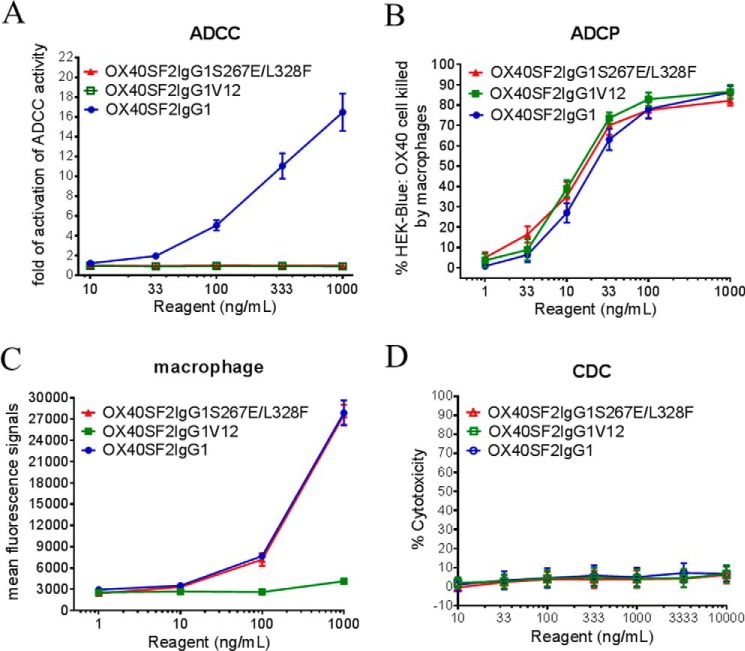
**Effector functions of anti-OX40 antibodies with S267E/L328F and V12 mutations.** *A,* increasing concentrations (10 to 1,000 ng/ml) of OX40SF2IgG1S267E/L328F, OX40SF2IgG1V12, and OX40SF2IgG1 were incubated with HEK-Blue:OX40 cells co-cultured with effectors cells and ADCC reporter bioassays were performed. The fold of activation of ADCC activities were plotted against the concentrations of test antibodies (data were presented as mean ± S.E., *n* ≥ 6). *B,* increasing concentrations (1 to 1,000 ng/ml) of OX40SF2IgG1S267E/L328F, OX40SF2IgG1V12, and OX40SF2IgG1 were incubated with GFP positive HEK-Blue:OX40 cells co-cultured with differentiated macrophages and the phagocytosis of GFP positive target cells were evaluated by flow cytometry assay. The percentages of GFP positive HEK-Blue:OX40 cells eliminated, which reflected the ADCP activities, were plotted against the concentrations of test antibodies (data were presented as mean ± S.E., *n* ≥ 6). *C,* increasing concentrations (1 to 1,000 ng/ml) of OX40SF2IgG1, OX40SF2IgG1S267E/L328F, and OX40SF2IgG1V12 antibodies were assessed for their binding to differentiated macrophages by flow cytometry assays. Mean fluorescence signals were plotted against the concentrations of test antibodies (data were presented as mean ± S.E., *n* = 2). *D,* increasing concentrations (10 to 10,000 ng/ml) of OX40SF2IgG1, OX40SF2IgG1S267E/L328F, and OX40SF2IgG1V12 antibodies were incubated with HEK-Blue:OX40 cells in the presence of rabbit complement. The CDC activities were quantitated by measuring lactate dehydrogenase (*LDH*) activity released from the cytosol of lysed HEK-Blue:OX40 cells and expressed as percent cytotoxicity relative to that lysed by Triton X-100 (data were presented as mean ± S.E., *n* = 7).

A bioluminescent reporter gene expression in effector cells served as a model of FcγRIIIA-mediated ADCC activation. When HEK-Blue:OX40 target cells were co-cultured with effector cells expressing FcγRIIIA, OX40SF2IgG1 activated reporter gene expression in a dose-dependent manner. Neither OX40SF2IgG1S267E/L328F nor OX40SF2IgG1V12 induced reporter gene expression (Fig. 4*A*), indicating that the S267E/L328F and V12 mutations abrogated the ADCC activity of OX40SF2IgG1 antibody. These results were consistent with the loss of binding activities for the engineered antibodies to FcγRIIIA (Fig. 2*D*).

The ADCP activities of the anti-OX40 antibodies with S267E/L328F and V12 mutations were evaluated via phagocytosis of GFP-expressed HEK-Blue:OX40 cells by macrophages differentiated from isolated monocytes. Both the OX40SF2IgG1S267E/L328F and the OX40SF2IgG1V12 had similar ADCP activities on HEK-Blue:OX40 target cells as OX40SF2IgG1 (Fig. 4*B*). The binding of engineered anti-OX40 antibodies to differentiated macrophages were assessed by a flow cytometry assay. The OX40SF2IgG1S267E/L328F antibody had binding affinity to macrophages similar to that of the OX40SF2IgG1 antibody. Although active in ADCP assay, the OX40SF2IgG1V12 antibody had a significantly reduced binding to macrophages (Fig. 4*C*).

The CDC activity was determined by a rabbit complement-mediated cell killing assay. The OX40SF2IgG1 antibody did not lead to significant CDC activity toward HEK-Blue:OX40 target cells up to 10,000 ng/ml. Likewise, neither the V12 nor S267E/L328F mutations facilitated higher CDC activities relative to the antibody with the native human IgG1 Fc domain (Fig. 4*D*).

##### Multimerization at the Cell Surface for Anti-OX40 Antibodies with Mutations That Promote IgG Hexamerization

**FIGURE 5.**
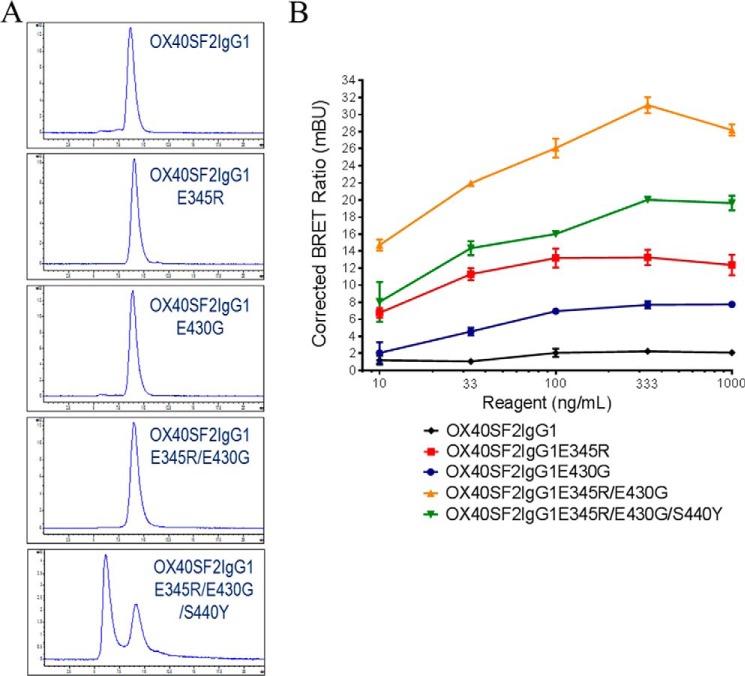
**Multimerization of anti-OX40 antibodies with the hexamerization mutations.** *A,* SEC profiles of OX40SF2IgG1, OX40SF2IgG1E345R, OX40SF2IgG1E430G, OX40SF2IgG1E345R/E430G, and OX40SF2IgG1E345R/E430G/S440Y antibodies. The *y* axes are absorbance at 280 nm (mAU) and the *x* axes are retention times (min). *B,* nanoBRET PPI assay for anti-OX40 antibodies with mutations that promoted IgG hexamerization. Increasing concentrations (from 10 to 1000 ng/ml) of both Nanoluc donor antibody and Halotag receptor antibody were applied to HEK-Blue:OX40 cells and NanoBRET PPI assays were conducted. Mean corrected BRET ratio were plotted against the concentrations of test antibodies (data were presented as mean ± S.E., *n* ≥ 2).

Diebolder *et al.* (23) identified a set of Fc mutations (E345R, E430G, S440Y) that can facilitate hexamerization of IgG1 Abs when bound to cell surface antigens. We hypothesized that antibody multimerization could enhance the agonism of therapeutic TNFR antibodies by facilitating the aggregation of TNFRs, which could result in receptor activation. To test this hypothesis, we engineered OX40SF2IgG1 antibody to have the E345R mutation (OX40SF2IgG1E345R), E430G mutation (OX40SF2IgG1E430G), E345R/E430G double mutations (OX40SF2IgG1E345R/E430G), and E345R/E430G/S440Y triple mutations (OX40SF2IgG1E345R/E430G/S440Y). SEC analyses revealed that OX40SF2IgG1E345R, OX40SF2IgG1E430G, and OX40SF2IgG1E345R/E430G antibodies were monomers in solution just as OX40SF2IgG1 (Fig. 5*A*). However, OX40SF2IgG1 antibody with E345R/E430G/S440Y triple mutations had a monomeric peak and a ∼900 kDa peak determined by SEC with multi-angle light scattering analysis (SEC-MALS), which was indicative of a hexamer state. This observation was consistent with the reported finding that E345R/E430G/S440Y mutations could promote hexamer formation readily in the solution phase (23).

To evaluate whether the engineered antibodies could multimerize upon binding antigens at the cell surface, a NanoBRET protein-protein interaction (PPI) proximity-based assay was developed to detect protein-protein interactions by measuring energy transfer from a bioluminescent protein donor to a fluorescent protein acceptor. SF2 antibodies with mutations that promote IgG oligomerization were further engineered to have either the Nanoluc or the Halo tags attached at the C termini of the light chains to serve as the donor and acceptor probes. The tagged antibodies showed comparable functional activities in HEK-Blue NF-κB reporter assays as the corresponding untagged antibodies (data not shown). NanoBRET PPI assays were performed by applying the donor and acceptor antibodies to HEK-Blue:OX40 cells. The association of multimerized antibodies were determined from calculation of the corrected NanoBRET ratios. Although OX40SF2IgG1 antibody had the background corrected NanoBRET ratio, SF2 Abs with the mutations that promoted IgG hexamerization showed much higher corrected NanoBRET ratios across the concentrations ranging from 10 to 1000 ng/ml (Fig. 5*B*). The degree of antibody association at the cell surface, reflected by the corrected NanoBRET ratio value, had the following rank order: OX40SF2IgG1E345R/E430G > OX40SF2IgG1E345R/E430G/S440Y > OX40SF2IgG1E345R > OX40SF2IgG1E430G.

##### Mutations That Promote IgG Hexamerization Enhanced Agonism Independent of FcγRIIB Cross-linking

**FIGURE 6.**
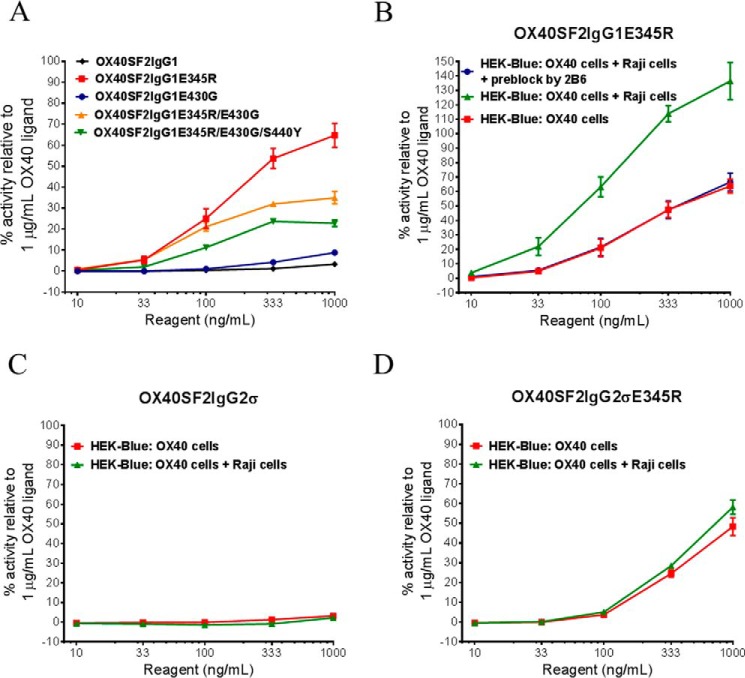
**HEK-Blue NF-κB reporter assay for the assessment of agonism of anti-OX40 antibodies with mutations that promoted IgG hexamerization.** *A,* increasing concentrations (10 to 1000 ng/ml) of OX40SF2IgG1, OX40SF2IgG1E345R, OX40SF2IgG1E430G, OX40SF2IgG1E345R/E430G, and OX40SF2IgG1E345R/E430G/S440Y antibodies were applied to HEK-Blue:OX40 cells and the agonistic activities of the antibodies were assessed by HEK-Blue NF-κB reporter assay. The agonistic activities of anti-OX40 antibodies, normalized as percent activity relative to that driven by 1 μg/ml of OX40 ligand, were plotted against the concentrations of test antibodies (data were presented as mean ± S.E., *n* ≥ 9). *B–D,* increasing concentrations (10 to 1000 ng/ml) of OX40SF2IgG1E345R (*B*), OX40SF2IgG2σ (*C*), and OX40SF2IgG2σE345R (*D*) antibodies were incubated with HEK-Blue:OX40 cells with or without co-culturing with Raji cells. In *B,* another set of assays were set up in which Raji cells were preincubated with 5 μg/ml of 2B6 antibody before co-culturing with HEK-Blue:OX40 cells to test the effect of blocking FcγRIIB cross-linking. The agonistic activities of the antibodies were assessed by the HEK-Blue NF-κB reporter assay. The agonistic activities of anti-OX40 antibodies, normalized as percent activity relative to that driven by 1 μg/ml of OX40 ligand, were plotted *versus* the concentrations of test antibodies (data were presented as mean ± S.E., *n* = 8).

To assess whether multimerized anti-OX40 antibody with hexamerization mutations had higher agonistic activity, the engineered antibodies were studied in HEK-Blue NF-κB reporter assay. Although the E345R mutation did not facilitate the highest antibody multimerization inferred from the NanoBRET PPI assay (Fig. 5*B*), OX40SF2IgG1E345R antibody led to the highest reporter gene expression in a dose-dependent manner (Fig. 6*A*). The anti-OX40 antibody with the E430G mutation had the lowest agonistic activity, whereas the antibodies with E345R/E430G double mutations and with E345R/E430G/S440Y triple mutations showed agonism better than that with E430G mutation but lower than that with the E345R mutation (Fig. 6*A*).

Because Glu^345^ is a conserved residue among IgG subtypes, we generated a E345R mutation into the same anti-OX40 Ab with a human IgG2 Fc domain with the Fc silencing mutations (OX40SF2IgG2σ) (31). HEK-Blue NF-κB reporter assay revealed that although OX40SF2IgG2σ had little agonistic activity, the OX40SF2IgG2σE345R showed agonism in a dose-dependent manner (Fig. 6). Hence the E345R mutation could enhance agonism without engagement of Fcγ receptors.

##### FcγRIIB Cross-linking Boost of the Agonism of Anti-OX40 Antibody with E345R Mutation Depending on the IgG Subtype

Although E345R mutation could increase the agonism of anti-OX40 antibody with either IgG1 or IgG2σ Fc independent of FcγRIIB cross-linking, the effect of FcγRIIB cross-linking on agonism was tested. The HEK-Blue NF-κB reporter assay was adapted to apply the engineered antibodies to HEK-Blue:OX40 cells co-cultured with Raji cells. The presence of Raji cells could boost the agonism of the OX40SF2IgG1E345R antibody over 2-fold (Fig. 6*B*). When 2B6 antibody was added to pre-block FcγRIIB receptor on Raji cells, the Raji cell-mediated boost of agonism for OX40SF2IgG1E345R was completely abrogated. This result demonstrated that the boost of agonism was driven by FcγRIIB cross-linking.

Similar assays were set up to evaluate FcγRIIB cross-linking on the agonism of OX40SF2IgG2σ antibody with or without the E345R mutation. For these constructs, the presence of Raji cells failed to boost the agonistic activity of either OX40SF2IgG2σ or OX40SF2IgG2σE345R antibody (Fig. 6, *C* and *D*). This data indicated that Raji cell mediated boost of agonism for anti-OX40 antibody with the E345R mutation depended on the silencing nature of IgG Fc.

##### Fc Effector Functions for Anti-OX40 Antibodies with E345R Mutation

**FIGURE 7.**
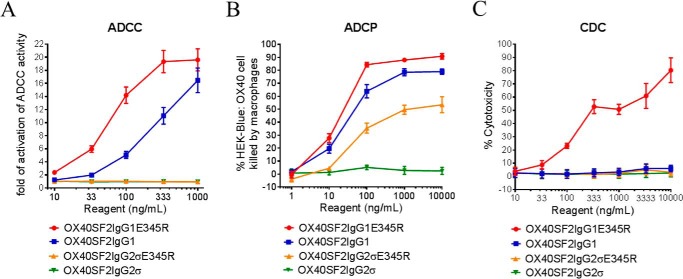
**Effector functions of anti-OX40 antibodies with the E345R mutation.** *A,* ADCC activities of anti-OX40 antibodies with E345R mutation. Increasing concentrations (10 to 1,000 ng/ml) of OX40SF2IgG1, OX40SF2IgG1E345R, OX40SF2IgG2σ, and OX40SF2IgG2σE345R were incubated with HEK-Blue:OX40 cells co-cultured with effectors cells and the ADCC reporter bioassays were performed. The folds of activation of ADCC activities were plotted against the concentrations of test antibodies (data were presented as mean ± S.E., *n* ≥ 4). *B,* increasing concentrations (1 to 10,000 ng/ml) of OX40SF2IgG1, OX40SF2IgG1E345R, OX40SF2IgG2σ, and OX40SF2IgG2σE345R antibodies were incubated with GFP positive HEK-Blue:OX40 cells co-cultured with differentiated macrophages and the phagocytosis of GFP positive target cells were evaluated by flow cytometry assay. The percentages of GFP positive HEK-Blue:OX40 cells eliminated, which reflected the ADCP activities, were plotted against the concentrations of test antibodies (data were presented as mean ± S.E., *n* ≥ 4). *C,* increasing concentrations (10 to 10,000 ng/ml) of OX40SF2IgG1, OX40SF2IgG1E345R, OX40SF2IgG2σ, and OX40SF2IgG2σE345R antibodies were incubated with HEK-Blue:OX40 cells in the presence of rabbit complement. The CDC activities were quantitated by measuring LDH activity released from the cytosol of lysed HEK-Blue:OX40 cells and expressed as percent cytotoxicity relative to that lysed by Triton X-100 (data were presented as mean ± S.E., *n* = 6).

The ADCC activities of the anti-OX40 antibodies with the E345R mutation were studied by the FcγRIIIA-mediated ADCC reporter bioassay. Although OX40SF2IgG1 antibody had ADCC activity, the OX40SF2IgG1 antibody with E345R mutation had more potent ADCC activity (Fig. 7*A*). In contrast, OX40SF2IgG2σ antibody did not have ADCC activity in this assay due to the silent Fc effector function property of this set of mutations. Likewise, the E345R mutation on OX40SF2IgG2σ antibody did not change its silencing in ADCC activity.

The ADCP activities of the anti-OX40 antibodies with E345R mutation were also studied by the phagocytosis of GFP-expressed HEK-Blue:OX40 cells by differentiated macrophages. Although the OX40SF2IgG1 antibody could dose-dependently mediate efficient killing of HEK-Blue:OX40 target cells by the macrophages, the E345R mutation only marginally enhanced the ADCP activity of OX40SF2IgG1 antibody (Fig. 7*B*). In contrast, whereas OX40SF2IgG2σ antibody did not show ADCP activity in this assay, the E345R mutation facilitated elevated ADCP activity of the OX40SF2IgG2σE345R antibody.

The CDC activities of the engineered anti-OX40 antibodies were studied by a complement mediated cell killing assay. Although OX40SF2IgG1 antibody did not mediate significant CDC activity toward HEK-Blue:OX40 target cells, the E345R mutation dose-dependently facilitated OX40SF2IgG1 antibody with higher CDC activity (Fig. 7*C*). In contrast, the OX40SF2IgG2σ antibody did not have CDC activity in this assay, and the E345R mutation on OX40SF2IgG2σ antibody did not change its silencing in CDC activity.

##### Human IgG2 Hinge Did Not Impart Agonistic Activity to Anti-OX40 Antibody

**FIGURE 8.**
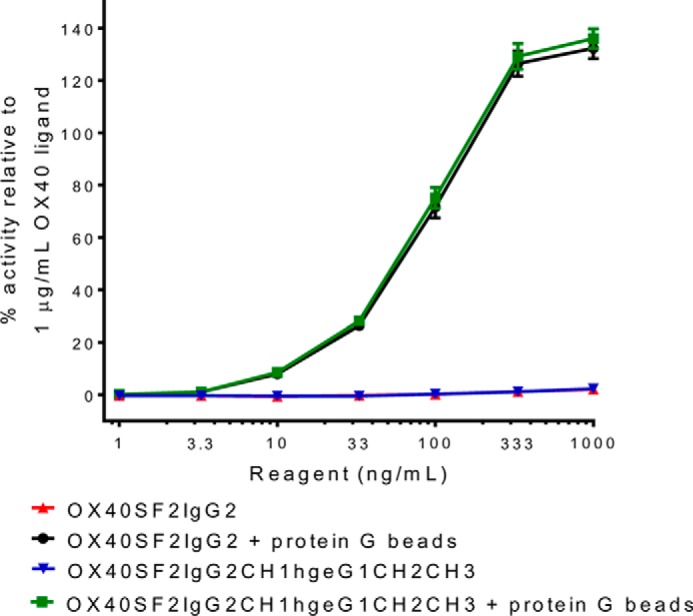
**HEK-Blue NF-κB reporter assay for the assessment of agonism of anti-OX40 antibodies with the human IgG2 hinge.** Increasing concentrations (1 to 1000 ng/ml) of OX40SF2IgG2 and OX40SF2IgG2CH1hgeG1CH2CH3 antibodies in the absence or presence of protein G beads were incubated with HEK-Blue:OX40 cells and their agonistic activities were assessed by HEK-Blue NF-κB reporter assay. The agonistic activities of anti-OX40 antibodies, normalized as percent activity relative to that driven by 1 μg/ml of OX40 ligand, were plotted against the concentrations of test antibodies (data were presented as mean ± S.E., *n* = 4).

White *et al.* (22) discovered that human IgG2 hinge can impart superagonistic activity to anti-CD40, 4-1BB, and CD28 antibodies independent of FcγRIIB cross-linking. To study whether this antibody engineering approach could apply to an anti-OX40 antibody, we generated the SF2 antibody with the IgG2 Fc (OX40SF2IgG2) and with a chimeric IgG Fc composed of the CH1 domain and hinge from IgG2 with the CH2 and CH3 domains from IgG1 (OX40SF2IgG2CH1hgeG1CH2CH3). By HEK-Blue NF-κB reporter assay, it was observed that neither OX40SF2IgG2 nor OX40SF2IgG2CH1hgeG1CH2CH3 showed agonistic activity when applied to HEK-Blue:OX40 cells (Fig. 8). Although the antibodies in solution showed little agonistic activity, both OX40SF2IgG2 and OX40SF2IgG2CH1hgeG1CH2CH3 could stimulate reporter gene expression dose-dependently to a level better than OX40 ligand at 1000 ng/ml when immobilized on protein G beads. Thus these IgG2 antibodies could be functional agonist molecules only upon cross-linking. These observations indicated that FcγR-independent agonism enhancement driven by the IgG2 hinge did not apply to the anti-OX40 SF2 antibody.

## Discussion

Agonist antibodies directed against immunostimulatory TNFRSFs are emerging as promising drugs for cancer therapy. Several recent Fc engineering efforts were gearing toward optimizing the antibodies for their antitumor immunity, with a focus on enhancing their agonistic activities. In this article, we applied these Fc engineering approaches to an anti-OX40 antibody and studied the effects of Fc mutations on binding to Fcγ receptors, on the agonistic activity, and on the ADCC, ADCP, and CDC effector functions.

To evaluate the agonistic activity of engineered antibodies, a HEK-Blue NF-κB reporter assay was developed to quantitate OX40 activation of the NF-κB pathway in a TNF receptor-associated factor-dependent manner (32, 33). The reporter gene expression could be stimulated by either OX40 ligand or OX40 IgG1 antibody in the immobilized state. Compared with the peripheral blood mononuclear cell-based assays that could have variability based on donor cell pre-activation state, this assay was robust with a high signal to background ratio and was amenable to a high throughput format. More importantly, by co-culturing Raji cells with HEK-Blue:OX40 cells, this reporter assay could be adapted to study the trans-acting effect of cross-linking to FcγRIIB expressed on Raji cells to the agonistic activities of the engineered anti-OX40 antibodies.

Two Fc engineering approaches were known to enhance the agonism of immunomodulatory antibodies against TNFRSFs by optimizing the antibody engagement with FcγRIIB receptor (20, 21). When applied to the anti-OX40 antibody, the reported S267E/L328F and V12 mutations could potently facilitate the binding of engineered anti-OX40 antibodies to FcγRIIB expressed in either transfected Expi293F cells or Raji cells. As a result, the engineered anti-OX40 antibodies showed agonism only when Raji cells were co-cultured with HEK-Blue:OX40 cells. Because pre-blocking of the FcγRIIB receptors on Raji cells significantly abrogated the Raji cell-dependent agonism enhancement, the agonistic activities of the engineered antibodies were mediated by FcγRIIB cross-linking. The enhancements of agonism were comparable for both the S267E/L328F and V12 constructs. The enhancement effects were very dramatic with over 2-fold better efficacy than the OX40 ligand by this reporter assay. The agonism enhancements mediated by FcγRIIB cross-linking were not driven by differential antibody internalizations because comparable amounts of OX40 antibodies with engineered Fc and native IgG1 remained bound on the OX40 expressing cells after the reporter assays (data not shown).

Besides the S267E/L328F and V12 mutations, we demonstrated that hexamerization mutations reported by Diebolder *et al.* (23) could also facilitate the enhancement of the agonism of anti-OX40 antibody but in a way not dependent on FcγRIIB cross-linking. Although anti-OX40 antibody with E345R, E430G, and E345R/E430G double mutations were monomers in solution, they become multimerized upon binding to OX40 receptors on the cell surface as shown by the NanoBRET protein-protein interaction assay. The multimerized antibodies showed enhanced agonistic activity in the absence of FcγRIIB-expressing cells, presumably due to the facilitation of increased clustering of OX40 receptors. The E345R mutation-mediated agonism enhancement also applied to OX40 antibody with the silent IgG2σ Fc, making it less likely that agonism enhancement was due to increased engagement of a serum factor to the IgG1 Fc domain. However, compared with mutations that enhanced FcγRIIB cross-linking, the agonism facilitated by the hexamerization mutation appeared to be moderate. In addition, although the double and triple mutations containing both E345R and E430G mutations facilitated higher antibody multimerization than any single mutation alone, the anti-OX40 antibody with the E345R single mutation showed the highest agonistic activity. In contrast, the combinations with the E430G mutation had lower agonism. Perhaps the oligomerized antibody facilitated by the E345R mutation had more favorable configuration in promoting the clustering of OX40 receptors than that with the E430G mutation.

Although E345R hexamerization mutation could facilitate agonism enhancement independent of FcγRIIB cross-linking, it was found that the presence of FcγRIIB-expressing cells could facilitate even higher agonism on OX40SF2IgG1E345R. Two observations indicated that the further boost of agonism depended on the engineered antibody interaction with FcγRIIB expressed on Raji cells. First, the boost of agonism was only observed on an antibody with engineered IgG1 Fc, which had binding affinity to FcγRIIB, but not with an antibody with the silent IgG2σ Fc. Second, the boost of agonism could be completely reversed by pre-blocking FcγRIIB expressed on Raji cells. It was reported that the multimerized antibody had much higher affinity to Fcγ receptors compared with a monomeric antibody in solution (31). Indeed, we observed by flow cytometry assay that the OX40SF2E345R/E430G/S440Y antibody, which had hexamer states in solution, had a more potent binding to FcγRIIB expressed on transfected Expi293F cells, compared with OX40SF2IgG1 and OX40SF2IgG1E345R antibodies, which were monomers in solution (data not shown). Thus the oligomerized OX40SF2IgG1E345R antibody bound to OX40 receptors on the cell surface could have increased binding to FcγRIIB on Raji cells, which in turn further stabilized antibody multimerization and facilitated receptor clustering that lead to the boost of agonism.

We also evaluated the Fc engineering approach claiming that a unique configuration of disulfide bonds in the hinge region of the IgG2 subtype could confer agonistic activity to immunostimulatory anti-TNFR antibodies independent of FcγRIIB cross-linking (22). However, the anti-OX40 SF2 antibody with CH1 domain and hinge from IgG2 Fc did not show elevated agonistic activity. Exactly how the IgG2 hinge confers agonist activity is not clear (22), and our observations suggested that the utility of this approach may depend on unique interaction of antibody to the specific epitope or specific target receptor.

Besides the agonism, the effects of Fc engineering on the effector functions of the engineered anti-OX40 antibody were also evaluated. ADCC is largely mediated by FcγRIIIA expressed on natural killer cells. The binding assays revealed that S267E/L328F and V12 mutations significantly disrupted the engineered anti-OX40 antibodies binding to FcγRIIIA while enhancing their binding to FcγRIIB, which is consistent with work reported from Mimoto *et al.* (21). As a consequence, it was observed that S267E/L328F and V12 mutations completely abrogated the ADCC effector functions of the engineered anti-OX40 antibodies. On the other hand, the E345R hexamerization mutation significantly further boosted the potency of ADCC activity of the engineered anti-OX40 antibody. The effect was specific for engineered antibody with IgG1 Fc, which was capable of binding to FcγRIIIA, but not for the antibody with the IgG2σ Fc, which did not bind to FcγRIIIA (Fc silent). These observations implied that the E345R-mediated boost of ADCC activity for IgG1 antibody was likely through increased binding of FcγRIIIA with oligomerized antibodies upon recognizing OX40 receptors on the cell surface.

Relative to anti-OX40 antibody with native IgG1 Fc, neither S267E/L328F nor the V12 mutations significantly changed the ADCP activities. This might be unexpected for the S267E/L328F mutations, which was reported to have enhanced binding to FcγRIIA (21), a major Fc receptor expressed on macrophages mediating phagocytosis. However, besides FcγRIIA, several Fc receptors, including FcγRI and FcγRIIIA, contribute to IgG antibody-mediated phagocytosis of target cells (34). The OX40SF2IgG1S267E/L328F antibody had similar binding potency to FcγRI as OX40SF2IgG1 but abrogated binding to FcγRIIIA. Besides, the degree of enhanced FcγRIIA binding was just to a level comparable with its binding to the high affinity FcγRI receptor. As a result, OX40SF2IgG1S267E/L328F antibody showed similar binding potency to macrophage as OX40SF2IgG1 and it did not show enhanced ADCP activity relative to OX40SF2IgG1. In contrast, the OX40SF2IgG1V12 antibody had reduced binding to FcγRI and FcγRIIIA, and unchanged binding potency to FcγRIIA, which might explain its significantly reduced binding to macrophages. Nonetheless, the OX40SF2IgG1V12 antibody showed a similar ADCP activity as OX40SF2IgG1. The reason for this discrepancy was unknown; perhaps the binding assays performed did not reflect the real binding affinity between the target-antibody immune complex and Fc receptors during the ADCP process. Similarly, no significant increase in ADCP activity was observed for OX40SF2IgG1 antibody with E345R mutation, although such a mutation significantly enhanced the ADCC activity of the engineered antibody. Interestingly, although OX40SF2IgG2σ did not have ADCP activity, the E345R mutation conferred significant ADCP activity to this antibody with silent Fc.

In the classical pathway of complement-dependent cytotoxicity, the binding of C1q to the antigen-antibody immune complex triggers the initiation of complement cascade that leads to the killing of target cells. The SF2 antibody with native IgG1 Fc did not have activity in the CDC assay. The S267E/L328F and V12 mutations, which facilitate antibody binding to FcγRIIB, apparently showed no effect on CDC activity. However, the E345R mutation, which promotes antibody hexamerization, facilitated significantly elevated CDC activity to SF2 antibody with IgG1 Fc but not with the silent IgG2σ Fc. This observation was corroborated to what was reported by Diebolder *et al.* (23), providing another example that hexamerized antibody may promote multivalent C1q binding and thus facilitate enhanced CDC activity.

In summary, each of the several Fc engineering approaches evaluated in this study offered a unique property to enhance the agonism and effector functions. The adoption of which approach depends on the specific TNFRSF target and on the individual paratope-epitope engagement, and many factors need to be considered. One major consideration is whether the ADCC effector function is required for the therapeutic activity of the antibody. For TNFRSF antibodies that require agonist activity with minimal ADCC activities, such as antibodies against CD40 and CD27, the S267E/L328F and V12 mutations can facilitate high agonism enhancement with abrogated ADCC activity. E345R mutation on a silent IgG Fc could also be a choice for a modest agonism enhancement. In contrast, for those targets that ADCC activity is required to eliminate regulatory T cells, such as anti-OX40 and anti-GITR antibodies, E345R mutation on IgG1 can facilitate enhanced ADCC and CDC activities besides enhanced agonistic activity. Another consideration is whether the dependence on FcγRIIB cross-linking is desired or not for agonism enhancement. The E345R hexamerization mutation can facilitate higher agonism independent of FcγRIIB cross-linking, which may equip antibody with defined therapeutic activity regardless of FcγR expression levels in the local microenvironment, particularly an advantage for those tumor microenvironments with low levels of infiltration of FcγR expressing cells. However, the non-dependence of FcγRIIB cross-linking may stimulate agonism non-specifically, which may lead to undesired off-target effects. In such case, the S267E/L328F or V12 mutations may be a better choice. Other factors, including the altered binding activities to different Fc receptors, the immunogenicity, PK profile, and developability of the engineered antibody should also be considered in the choice of the optimal engineering approach. In this study, we evaluated these engineering approaches side-by-side using *in vitro* assays, which laid a good foundation for further studies using primary cells, *in vivo* animal studies, and clinical studies to evaluate these approaches more rigorously.

## Experimental Procedures

### Fc Engineering of Anti-OX40 Antibody

Plasmids encoding the heavy chain (HC) and light chain (LC) of a humanized anti-OX40 antibody SF2 (27), were constructed for the expression of SF2 antibody with human IgG1 Fc (OX40SF2IgG1), IgG2 Fc (OX40SF2IgG2), or IgG2σ Fc (OX40SF2IgG2σ) domains (31). Gene syntheses were performed by Genewiz (South Plainfield, NJ) to introduce further mutations (EU numbering) on Fc of the heavy chain constructs to express the following engineered anti-OX40 antibodies described in this study.

#### 

##### OX40SF2IgG1S267E/L328F

OX40SF2IgG1 antibody with mutations S267E and L328F in the human IgG1 Fc domain.

##### OX40SF2IgG1V12

OX40SF2IgG1 antibody with mutations E233D, G237D, P238D, H268D, P271G, and A330R in the human IgG1 Fc domain.

##### OX40SF2IgG1E345R

OX40SF2IgG1 antibody with mutation E345R in the human IgG1 Fc domain.

##### OX40SF2IgG2σE345R

OX40SF2IgG2σ antibody with mutation E345R in the human IgG2σ Fc domain.

##### OX40SF2IgG1E430G

OX40SF2IgG1 antibody with mutation E430G in the human IgG1 Fc domain.

##### OX40SF2IgG1E345R/E430G

OX40SF2IgG1 antibody with mutations E345R and E430G in the human IgG1 Fc domain.

##### OX40SF2IgG1E345R/E430G/S440Y

OX40SF2IgG1 antibody with mutations E345R, E430G, and S440Y in the human IgG1 Fc domain.

##### OX40SF2IgG2CH1hgeG1CH2CH3

OX40SF2IgG2 antibody with the CH2 and CH3 domains swapped from those of human IgG1 Fc domains.

### Antibody Expression and Purification

Plasmids encoding antibody HC and LC were co-transfected at a 1:3 (HC:LC) molar ratio into Expi293F cells following the transfection kit instructions (Thermo Scientific, San Jose, CA). Cells were spun down 5 days post-transfection and the supernatant passed through a 0.2-μm filter. The titer of antibody expression was quantified using Octet (ForteBio, Menlo Park, CA). Antibody purification was carried out using pre-packed Protein A spin columns following the kit instructions (GE Healthcare Life Sciences). The purified antibody was buffer-exchanged into Dulbecco's PBS, pH 7.2, by dialysis and protein concentration was determined by UV absorbance at 280 nm. Quality was assessed by high-performance SEC and SDS-PAGE of reduced and non-reduced samples.

### HEK-Blue NF-κB Reporter Assay

A stable HEK-Blue reporter cell line expressing human OX40 (HEK-Blue:OX40) was established by transfection of an OX40 expression plasmid (pUNO1-hOX40) into HEK-Blue null 1_v cells (InvivoGen, San Diego, CA) followed by the selection of stable expression clones. For the HEK-Blue NF-κB reporter assay, 1 × 10^5^ HEK-Blue:OX40 cells resuspended in 200 μl of culture media were aliquoted in each well of the 96-well assay plate and the OX40 ligand or anti-OX40 antibodies were added. To test the cross-linking effect, either 1 μl of protein G magnetic beads (Thermo Scientific, San Jose, CA) or 1 × 10^5^ Raji cell was added in the same assay well. After incubation at 37 °C overnight, the agonistic activities of the antibodies were evaluated by the quantification of the induced SEAP reporter gene expression using Quanti-Blue detection kit (Invivogen). Briefly, 40 μl of cell culture supernatant was mixed with 160 μl of Quanti-Blue reagent and incubated at 37 °C until the appropriate blue color developed. The OD at 650 nm was measured using a SpectraMax microplate reader (Molecular Devices, Sunnyvale, CA). The agonistic activity of anti-OX40 antibody was normalized as percent activity relative to that induced by 1 μg/ml of OX40 ligand.

### NanoBRET Protein-Protein Interaction Assay

The coding sequence for the light chain of anti-OX40 SF2 antibody was cloned into pNLF-C and pHTC halotag vectors (Promega, Madison, WI) in-frame with C-terminal Nanoluc and Halotag sequences, respectively. These light chains were paired with the heavy chains for OX40SF2IgG1, OX40SF2IgG1E345R, OX40SF2IgG1E345R/E430G, and OX40SF2IgG1E345R/E430G/S440Y antibodies to express Fc engineered SF2 antibodies with either Nanoluc or Halotag attached at the C termini of the light chains. Standard Protein A spin column were employed to purify these modified antibodies.

To study antibody multimerization on the cell surface by the NanoBRET protein-protein interaction assay (Promega, Madison, WI), 0.25 × 10^5^ HEK-Blue:OX40 cells were seeded in each well of the 96-well assay plate and cultured at 37 °C overnight. The next day, equal concentrations of Nanoluc-tagged antibody (donor) and Halotag-tagged antibody (acceptor) in 50 μl of assay medium (Opti-MEM I reduced serum medium, no phenol red plus 4% FBS) were applied to the cells. Halotag 618 ligand diluted 1:1000 in 50 μl of assay medium were added in experimental well, and a no ligand control well was also set up by diluting DMSO 1:1000 in assay medium. After incubation at 37 °C for 30 min, the cells were washed twice and re-suspended in 100 μl of assay medium. 25 μl of Nano-Glo substrate, diluted 1:200 in assay medium without FBS, was added to each well. After shaking for 30 s, the donor emission (460 nm) and acceptor emission (618 nm) were measured by Envision. Raw NanoBRET ratio values with milliBRET units (mBU) were calculated as RawBRET = 618 nm_Em_/460 nm_Em_ × 1000. To factor in donor-contributed background or bleed through, corrected NanoBRET ratio values with milliBRET units were calculated as corrected BRET = RawBRET of experimental sample − RawBRET of no-ligand control sample, which reflected energy transfer from a bioluminescent protein donor to a fluorescent protein acceptor due to protein-protein interactions.

### Flow Cytometry Staining

Plasmids expressing human FcγRI (NM_000566), FcγRIIA (NM_021642), FcγRIIB (NM_004001), and FcγRIIIA (NM_000569) (Origene Technologies, Rockville, MD) were transiently transfected into Expi293F cells by Expifectmine293 transfection kit (Thermo Scientific). Flow cytometry assays were performed 48 h after transfection. To confirm the expression of transfected Fc receptors, their specific antibodies, 10.1 (BD Pharmingen) for FcγRI, IV.3 (StemCell Technologies, Vancouver, Canada) for FcγRIIA, 2B6 (in house preparation) for FcγRIIB (30), and 3G8 (BD Pharmingen) for FcγRIIIA, were employed in flow cytometry staining as positive controls. Raji cells (ATCC: CCL-86) were also employed to test the binding of anti-OX40 antibody to FcγRIIB receptor.

2 × 10^5^ cells per well were seeded in 96-well plate and blocked in BSA Stain Buffer (BD Biosciences) for 30 min at 4 °C. Cells were incubated with test antibody on ice for 1.5 h at 4 °C. After being washed twice with BSA stain buffer, the cells were incubated with R-phycoerythrin-labeled anti-human or anti-mouse IgG secondary antibody (Jackson ImmunoResearch Laboratories, West Grove, PA) for 45 min at 4 °C. The cells were washed twice in stain buffer and then re-suspended in 150 μl of Stain Buffer containing 1:200 diluted DRAQ7 live/dead stain (Cell Signaling Technology, Danvers, MA). PE and DRAQ7 signals of the stained cells were detected by Miltenyi MACSQuant flow cytometer (Miltenyi Biotec, Bergisch Gladbach, Germany) using B2 and B4 channels, respectively. Live cells were gated on DRAQ7 exclusion and the geometric mean fluorescence signals were determined for at least 10,000 live events collected. FlowJo software (Tree Star, Ashland, OR) was used for analysis. Data were plotted as the logarithm of antibody concentration *versus* mean fluorescence signals. Nonlinear regression analysis was performed by GraphPad Prism 6 (GraphPad Software, La Jolla, CA) and EC_50_ values were calculated.

### ADCC Assay

The ADCC activities of anti-OX40 antibodies were evaluated by an ADCC reporter bioassay as instructed by the manufacturer (Promega, Madison, WI). Briefly, 25,000 HEK-Blue:OX40 cells per well plated in a 96-well plate overnight were mixed with the engineered effector cells in which the activation of FcγRIIIA receptor lead to the expression of a luciferase reporter. Anti-OX40 antibodies were added to the cells and incubated at 37 °C for 6 h. Then Bio-Glo luciferase reagent was added and the luciferase signals were quantitated by Envision. The ADCC activities of anti-OX40 antibody were expressed as fold of activation of luciferase signals over that without testing antibody added.

### ADCP Assay

An OX40 target cell line expressing GFP was established by infecting HEK-Blue:OX40 cells with a Turbo GFP transduction particle (Sigma). Stable GFP-expressing cells were selected with puromycin. The human CD14^+^CD16^+^ monocytes were isolated from peripheral blood mononuclear cells (Biological Specialty, Colmar, PA) using a negative human monocyte enrichment kit without CD16 depletion (StemCell Technologies, Vancouver, Canada). Isolated monocytes were plated in X-VIVO 10 medium (Lonza, Basel, Switzerland) containing 10% FBS and macrophages were differentiated from monocytes by the addition of 25 ng/ml of macrophage colony-stimulating factor (R&D Systems, Minneapolis, MN) for 7 days. IFNγ (50 ng/ml; R&D Systems) was added for the final 24 h of differentiation. For the ADCP assay, 1 × 10^5^ cells/well differentiated macrophages were mixed with 0.25 × 10^5^ cells/well of GFP-expressing HEK-Blue:OX40 cells (4: 1 ratio) in 200 μl of medium (DMEM + 10% FBS) in 96-well U-bottom plates. The test antibodies were added and the plate was incubated in a 37 °C incubator for 24 h. Then the cells were detached using Accutase (Sigma) and re-suspended in BSA Stain Buffer. Macrophages were stained with anti-CD11b and anti-CD14 antibodies (BD Biosciences) coupled to Alexa Fluor 647 (Thermo Scientific). GFP positive HEK-Blue:OX40 target cells and Alexa 647 positive macrophages were identified by flow cytometry using Miltenyi MACSQuant flow cytometer (Miltenyi Biotec). The data were analyzed using FlowJo software (Tree Star) and ADCP-mediated cell killing was determined by measuring the reduction in GFP fluorescence using the following equation: percentage of target cells killed = ((percentage of GFP^+^, CD11b^−^, CD14^−^ cells with the lowest concentration of antibody) − (percentage of GFP^+^, CD11b^−^, CD14^−^ cells with the test concentration of antibody))/(percentage of GFP^+^, CD11b^−^, CD14^−^ cells with the lowest concentration of antibody) × 100.

### CDC Assay

The CDC activities of anti-OX40 antibodies were evaluated by a complement-mediated cell killing assay. Briefly, 1 × 10^5^ HEK-Blue:OX40 cells were incubated with 6.7% (v/v) rabbit complement (Cedar Lane Labs, Burlington, Canada) and testing antibodies for 1 h. The lactate dehydrogenase activity released from the cytosol of lysed HEK-Blue:OX40 cells into the supernatant were measured by a Cytotoxicity Detection Kit according to manufacturer's instructions (Roche Diagnostics, Indianapolis, IN). The complement-mediated cytotoxicity was expressed as percent cytotoxicity relative to that lysed by 0.67% (v/v) Triton X-100.

## Author Contributions

D. Z., M. G., and M. C. conceived the study. D. Z. performed the experiments, analyzed the data, and wrote the paper with M. C. All authors reviewed the results, contributed to the writing, and approved the final version of the manuscript.

## Footnotes

3 The abbreviations used are:

TNFRSF: tumor necrosis factor receptor superfamily
ADCC: antibody-dependent cellular cytotoxicity
ADCP: antibody-dependent cellular phagocytosis
BRET: bioluminescence resonance energy transfer
CDC: complement-dependent cytotoxicity
CTLA-4: cytotoxic T lymphocyte antigen 4
GITR: glucocorticoid-induced TNFR-related protein
HVEM: Herpesvirus entry mediator
PPI: protein-protein interaction
SEC: size-exclusion chromatography
SEAP: secreted embryonic alkaline phosphatase
HC: heavy chain
LC: light chain.
